# Centromeres Cluster De Novo at the Beginning of Meiosis in *Brachypodium distachyon*

**DOI:** 10.1371/journal.pone.0044681

**Published:** 2012-09-07

**Authors:** Ruoyu Wen, Graham Moore, Peter J. Shaw

**Affiliations:** John Innes Centre, Norwich Research Park, Norwich, United Kingdom; Oklahoma Medical Research Foundation, United States of America

## Abstract

In most eukaryotes that have been studied, the telomeres cluster into a bouquet early in meiosis, and in wheat and its relatives and in Arabidopsis the centromeres pair at the same time. In *Arabidopsis*, the telomeres do not cluster as a typical telomere bouquet on the nuclear membrane but are associated with the nucleolus both somatically and at the onset of meiosis. We therefore assessed whether *Brachypodium distachyon*, a monocot species related to cereals and whose genome is approximately twice the size of *Arabidopsis thaliana,* also exhibited an atypical telomere bouquet and centromere pairing. In order to investigate the occurrence of a bouquet and centromere pairing in *B distachyon*, we first had to establish protocols for studying meiosis in this species. This enabled us to visualize chromosome behaviour in meiocytes derived from young *B distachyon* spikelets in three-dimensions by fluorescent in situ hybridization (FISH), and accurately to stage meiosis based on chromatin morphology in relation to spikelet size and the timing of sample collection. Surprisingly, this study revealed that the centromeres clustered as a single site at the same time as the telomeres also formed a bouquet or single cluster.

## Introduction

Meiosis is a reductional cell division process in sexually reproducing eukaryotes that ensures gametes carry a halved number of chromosomes, so that subsequent fertilization, in which male and female gametes combine, results in the correct somatic chromosome number. Organisms exhibiting sexual reproduction carry two copies (homologues) of each chromosome. Homologues need to recognise and pair with their partner at the onset of meiosis ensuring correct segregation so that gametes only end up with a single copy of each chromosome. Certain organisms, including many plants, are polyploids, meaning that their genomes comprise more than one ancestral genome. This means that, as well as a true homologue, each chromosome has two or more closely related chromosomes termed homoeologues. In order for meiosis to segregate the chromosomes properly and a stable genome to be maintained, the homologues must be correctly distinguished from their homoeologues; effectively the genome must behave as a true diploid in pairing behaviour. The mechanism whereby the homologues and homoeologues are distinguished is a very active area of research, particularly in wheat, since the *Ph1* locus, which is the major locus in wheat controlling pairing specificity, has importance potential applications in plant breeding [1], [2]. Before meiosis, each homologue is replicated, forming two sister chromatids that remain linked together. At the start of meiosis, each chromosome must recognize its homologue from among all of the chromosomes present in the nucleus. In many organisms, this sorting process involves the telomeres of the chromosomes clustering on the nuclear membrane to form a bouquet at the onset of meiosis [3], [4], [5], [6], [7], [8]. Telomere clustering brings the telomeres of homologues into close proximity so that they can intimately associate. The homologues then generally become intimately aligned or paired along their entire lengths. As part of this alignment, a proteinaceous structure known as the synaptonemal complex (SC) is assembled between the associated chromosomes in a process termed synapsis (reviewed by Zickler and Kleckner [9]). The general view is that synapsis is initiated within the intimately associated telomere regions and then extends towards the centromeres. Meiotic recombination (the exchange of DNA strands) is completed within the synapsed structure, resulting in crossover formation between the DNA strands of the homologues thereby reshuffling genetic information. Meiotic recombination occurs through the generation and repair of double strand breaks (DSBs) using the homologues. Importantly, meiotic recombination forms chiasmata, physical links which together with sister chromatid cohesion hold the homologues together after the disassembly of the SC. This enables homologues to be correctly orientated and segregated on the first meiotic spindle so that each gamete carries only a single member of each pair of homologous chromosomes. A second division occurs in which the sister chromatids are segregated so that each gamete carries only a single copy of each chromosome [10].

Recent studies suggest that the initial sorting process is more complex in plants, involving more than just the telomeres. Centromeres in tetraploid and hexaploid wheat can associate in pairs during floral development prior to telomere bouquet formation [11], before clustering as 7 sites at telomere bouquet formation [12]. The occurrence of centromere pairing prior to the telomere bouquet in wheat indicates that the pairing process is independent of the telomere clustering and its subsequent pairing mechanism. Recently it has also been shown in *Arabidopsis* that the centromeres engage in pairing at the onset of meiosis [13]. Moreover this centromere pairing is not only independent of the telomere clustering, but synapsis is initiated at the centromere paired regions independently of the telomere pairing, since the homologous centromeres still synapse when intimate alignment and synapsis is disrupted between the chromosome arms [13].

In *Arabidopsis*, the telomeres do not cluster as a typical telomere bouquet on the nuclear membrane but are associated with the nucleolus both somatically and at the onset of meiosis [14]. We therefore assessed whether *Brachypodium distachyon* whose genome is approximately twice the size of *Arabidopsis thaliana* [15] also exhibited an atypical telomere bouquet and centromere pairing (see [16], [17] for recent reviews of Brachypodium architecture and cytogenetics). In order to investigate this, we first had to establish new protocols for studying meiosis in *B distachyon*. This enabled us to visualize chromosome behaviour in meiocytes derived from young *B distachyon* spikelets in three-dimensions by fluorescent in situ hybridization (FISH), and accurately to stage meiosis based on chromatin morphology in relation to spikelet size and the timing of sample collection. Surprisingly, this study revealed that the centromeres clustered as a single site at the same time as the telomeres also formed a bouquet or single cluster.

## Materials and Methods

### 1 Plant Growing

The *B. distachyon* plants were handled following methods previously described [18]. In detail, the top glumes of the seeds were removed, and seeds were then soaked in 10% sodium hypochlorite solution containing a drop of Tween-20 for 3 mins. The seeds were then rinsed in sterile water three times to remove sodium hypochlorite. Sterilised seeds were transferred to sterile 90 mm Petri dishes covered with two layers of damp sterile filter papers at a density of 15 seeds per Petri dish. Petri dishes were wrapped in foil and incubated at 4°C for two days to synchronise germination, followed by a week at 25°C with a 16 h photoperiod until shoots and roots were well formed (seeds were only incubated for 2 days at 25°C until roots were 0.5–1 cm long if they were to be used in metaphase spread preparation). Young seedlings were transferred, one plant per cell, into 40 cell trays containing Medicago mix (50% John Innes Compost No. 2 and 50% peat and grit mix). Plants were grown in a controlled environment room at 22°C with a 20 h photoperiod. After 2 weeks of growth in the tray, young plants were transferred into rootrainers and grown there until harvest.

### 2 Microtome Sectioning

After collection, young spikelets were immediately fixed in 4% formaldehyde, freshly prepared from paraformaldehyde, in 1×PBS with 0.01% Tween, pH7.0, at 4°C overnight. Fixed samples were washed with 1×PBS for 15 min and then processed in an automatic embedding machine (Tissue-tech VIP E300, SAKURA FINETEK, USA) with a standard wax embedding protocol (70%, 80% and 90% ethanol each 4 h at 35°C, then 3×4 h of 100% ethanol at 35°C, 3×4 h of 100% xylene at 35°C and 4×4 h of wax at 60°C). After embedding, samples were positioned in hot wax and then cooled down on a cold plate. Wax embedded samples were cut into 10 µm thick section strips with a microtome. Wax strips were flattened by distilled water on APTES treated slides [19] and then air dried at 42°C overnight. Wax was then removed by two rounds of 100% xylene wash, each 15 min. After being thoroughly dried in a fume hood, the specimens on the slides were treated for 2 h at 37°C with 30 µl of enzyme mixture (the same as used in metaphase chromosome spread preparation) in a humid chamber. After enzyme digestion, the slides were washed twice in 1×PBS and then dehydrated by a methanol series (30%, 50%, 70%, 100%, 2 mins each). After the slides were thoroughly dried in the fume hood for at least 3 h, borders around the specimens on the slides were drawn with a mini liquid blocker pen (Cosmo Bio, Japan). After the liquid blocker had dried, the slides were ready to be used for in situ hybridization.

### 3 Metaphase Chromosome Spread Preparation

Metaphase chromosome spread slides were prepared using the method described by [20] with the only major modification being the use of a modified enzyme mixture for the digestion of Brachypodium distachyon (Bd) roots in this study. This modified enzyme mixture (see below for details) worked better with the FISH protocol used in this study. Bd seeds were germinated as described above. Young seedlings having 0.5–1 cm length roots were immersed in iced water for 24 hr and then fixed in freshly prepared fixative (4% formaldehyde in 1×PBS with 0.01% Tween, pH7.0) at 4°C overnight. Fixed seedlings were transferred to a small watch glass and washed for 3×5 min in citrate buffer (100 mM citric acid monohydrate and 100 mM trisodium citrate dihydrate solution mixed at 4∶6 v:v, pH adjusted to 4.8 with one or other solution, then diluted 1∶10 with distilled water for use) to remove the fixative. Seedlings were dissected during the first wash to keep only the distal parts of the roots (about 3 mm long). Citrate buffer was drained thoroughly by clean filter paper, and the roots were treated at 37°C for one hour with 100 µl of enzyme mixture in citrate buffer (2% cellulase - Onozuka, R-10; 1% pectolyase - Sigma-Aldrich Co., cat. no. P-3026; 1% pectolyase-Y23 - Duchefa Biochemie B.V., cat. no. P8004). Inclusion of the two different sources of pectolyase gave better results than either alone, whereas inclusion of cytohelicase, often added in digestion mixes, had no effect in our experiments. After treatment, the enzyme mixture was carefully removed and digested roots were washed with cold citrate buffer on ice for 15 min. The roots were then carefully transferred to clean slides one by one, using a pipette with a truncated plastic tip. The roots were very soft and fragile at this stage, and normally only the root tips would be left after the transfer. Citrate buffer on the slides was carefully replaced by 8 µl of 45% acetic acid per slide and the slides were covered with 18×18 mm coverslips and put in a humid chamber for 15 mins. The coverslips were squashed firmly for a few seconds and then immediately frozen in liquid nitrogen for 1 min. The coverslips were then quickly flicked off with a scalpel blade and the slides were flooded a few times with Carnoy’s fixative (3∶1 ethanol to acetic acid) at −20°C. After the fixative was drained, the slides were immersed in 100% ethanol for 15 min and then air-dried. The air-dried slides were post-fixed in freshly prepared fixative (4% formaldehyde in 1×PBS with 0.01% Tween, pH7.0) at room temperature for 20 min and then washed with 1×PBS twice before being dehydrated in a methanol series (30%, 50%, 70%, 100%, 2 mins each). After the slides were thoroughly dried in a fume hood for at least 3 h, borders around the samples were drawn with a mini liquid blocker pen (Cosmo Bio, Japan). After the liquid blocker had dried, the slides were ready to be used for in situ hybridization.

### 4 Probe Labelling

Telomere DNA probe was produced by PCR using (TTAGGG)_5_ and (CCCTAA)_ 5_ as both primers and templates, a method first developed by Ijdo *et al* [21]. PCR products were purified by columns (QIAquick PCR Purification Kit, Qiagen, Germany) before being used as template in nick translation labelling.

1–1.5 µg DNA template was labelled by nick translation (Nick Translation Mix, Roche Diagnostics Ltd, Germany, Cat No. 11745808910) following the instructions provided by the manufacturers. Telomere DNA was labelled with Biotin-16-dUTP (Roche Diagnostics Ltd, Germany), and centromeric DNA was labelled with Digoxigenin-11-dUTP (Roche Diagnostics Ltd, Germany). Every 20 µl of nick translation labelling product was cleaned by standard DNA ethanol precipitation method and re-suspended in 10 µl of hybridization mixture (see in situ hybridization method for details) and stored at −20°C.

### 5 In situ Hybridization and Signal Detection

The in situ hybridization protocol was adapted from [19]. In brief, labelled probes were mixed with 10 times the volume of hybridization mixture (50% deionised formamide, 10 mM PIPES pH 8.0, 1 mM EDTA, 10% dextran sulfate, 30 mM NaCl, 50 µg/ml salmon sperm blocking DNA), denatured in boiling water for 8 min and chilled on ice for at least 5 min. 20–40 µl of denatured probe mixture was added to each slide and covered with a plastic cover slip. The slides were denatured in a thermo cycler (Hybaid OmniSlide, Thermo Fisher Scientific Inc.) at 83°C for 12 min if microtome section slides were used, or at 78°C for 8 min if chromosome spread slides were used. Slides were then cooled down progressively in the same thermo cycler (55°C for 3 min, 50°C, 42°C, 40°C, 38°C each 5 min and kept at 37°C). After the temperature reached 37°C, the slides were transferred into a humid chamber and incubated at 37°C overnight.

Cover slips were carefully removed in 2×SSC at 42°C the next day, and the slides were washed sequentially twice in 20% formamide, 0.1×SSC for 5 min at 42°C, twice in 2×SSC for 5 min at 42°C, once in 2×SSC for 5 min at RT and finally twice in 4×SSC, 0.2% Tween 20 for 5 min at RT. After the slides were briefly drained on tissue paper, 100 µl of blocking solution (5% BSA in 4×SSC, 0.2% Tween 20) was added to each slide and incubated for 5 min at 37°C. Then the blocking solution was replaced by 100 µl of detection solution and the slides were covered by plastic cover slips and incubated at 37°C for 45 min in a humid chamber. Sheep anti-digoxigenin-fluorescein (Roche Diagnostics Ltd, Germany) and/or ExtrAvidin-Cy3 (Sigma-Aldrich Co.) were diluted 1∶300 in blocking solution to make the detection solution for digoxigenin-11-dUTP and/or biotin-16-dUTP labelled probes respectively. After detection, the slides were washed three times in 4×SSC, 0.2% Tween 20 for 5 min at RT. Each specimen was finally mounted under a glass cover slip with 40 µl of Vectashield (Vector Laboratories Inc., USA, Cat. No. H-1000) containing 1µg/ml DAPI and the coverslips were fastened with nail polish at their corners before image acquisition.

### 6 Image Acquisition and Analysis

The images of centromere labelling on chromosome spreads were acquired by a Nikon Eclipse E600 epi-fluorescence microscope equipped with a Hamamatsu Orca-ER cooled CCD camera and a Prior Proscan xyz stage using MetaMorph (Universal Imaging) software. Images were analysed with ImageJ [22].

The image stacks of centromere and telomere labelling on microtome tissue sections were acquired by a Zeiss LSM510 confocal microscope with motorised stage and equipped with Violet Diode (405 nm), Argon Ion (457, 488, 514 nm), DPSS (561 nm) and Red HeNe (633 nm) lasers. Image stacks were analysed with ImageJ. 3D image projections were produced by using the VolumeJ plugin for ImageJ [23].

The scale bars of all the images were produced in ImageJ. Images were finally composed for publication using Adobe Photoshop CS3 (Adobe systems Inc.).

## Results

### 1 Staging of B. distachyon Meiosis in PMCs

**Figure 1:**
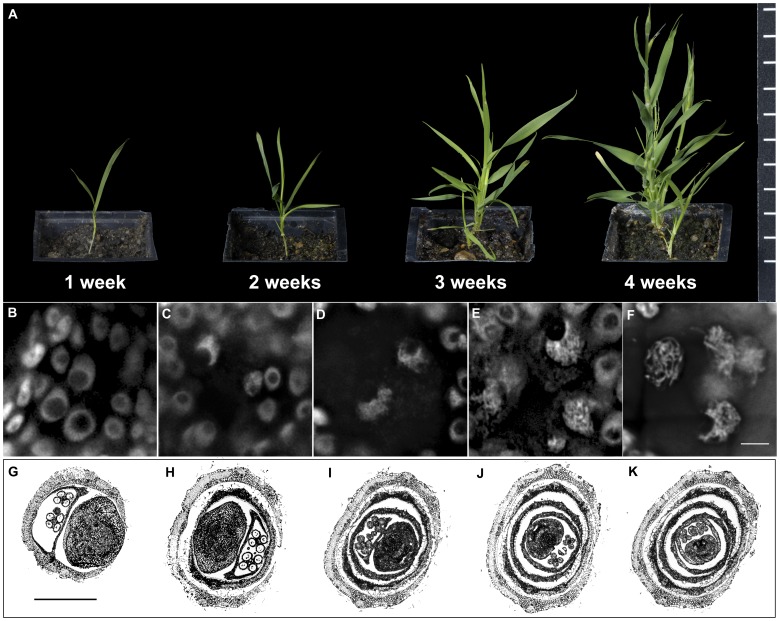
Staging of *B. distachyon* meiosis in PMCs. **A**: *B. distachyon* plants at different stages from young seedling to flowering. **B-F**: *B. distachyon* PMC meiocytes at different stages, stained with DAPI. Bar = 5 µm. **B**: PMCs at pre-meiosis stage: nuclei of PMCs at this stage are large, smooth and round; nucleoli are intact and located in the centre of the nuclei. **C**: Meiocytes at leptotene stage: thin chromatin threads start to appear in the nuclei of PMCs at this stage, but in general nuclei still look smooth; nucleoli move to the edges of the nuclei, and as a result the nuclei appear as crescent shapes; **D**: Meiocytes at zygotene stage: chromatin threads become thicker at this stage, which gives the nuclei a fluffy appearance; nucleoli are still at the edges of the nuclei, and some nuclei start to lose their clear boundaries. **E**: Meiocytes at late zygotene stage: thick chromatin threads become obvious and start to separate at this stage, which leaves dark regions in the nuclei; nucleoli are still at the edges of the nuclei. **F**: Meiocytes at pachytene stage: thick chromatin threads separate completely from each other at this stage; nuclei mostly appear as irregular shapes and nucleoli are dispersed in most nuclei. Meiocytes only remain for a very short time (probably 2 hr or less) at leptotene stage (**C**), and then change from zygotene (**D**) to late zygotene (**E**) stage gradually during a longer period (approximately 10 hr). Meiocytes quickly move from late zygotene (**E**) to pachytene (**F**) stage and stay at pachytene (**F**) for a few hours. After pachytene, it takes meiocytes only about 8 hr to go through all the remaining steps until the tetrad stage. **G–K**: Wax embedded sections of one *B. distachyon* spikelet at meiosis. Bar = 0.5 mm. **G** shows the image of the base floret. Images from **G** to **K** show florets in sequence from base to top of the spikelet. Florets on the same spikelet go through meiosis successively. Meiosis starts first in the base floret and last in the top floret with an interval of 8–10 hr. For instance, if meiocytes of the base floret (**G**) were at pachytene stage (**F**), the second from base floret (**H**) would be at about zygotene stage (**D**), and the third from base floret (**I**) would be at pre-meiosis stage (**B**).

Anther squashing is frequently used to prepare samples for studying meiosis. However the strong external physical force of squashing modifies the three-dimensional spatial structure of the nuclei and may produce artefacts [24]. Tissue thin sectioning is preferable because of its good maintenance of fine nuclear structures. Vibratome sectioning of young wheat spikelets was established in our lab previously [19], [24] and successfully applied in various chromosome behaviour studies in wheat [1], [11], [25]. However, due to the structure of *Brachypodium* spikelets (Fig. 1G–K), the florets are very likely to drop out during the process of sectioning, which makes it very difficult, if not impossible to section young Brachypodium spikelets with the original vibratome sectioning protocol. In order to overcome this problem we developed a modified protocol in which the trimmed spikelet was attached to the sectioning platform of the vibratome with super-glue, after which the entire spikelet was covered with a thin layer of super-glue. The super-glue was removed by treatment of the resulting sections with 50% (v/v) DMSO/water. This enabled good vibratome sections to be collected which produced good FISH results. However, even with the modified protocol, only the base floret of each spikelet was usable since the other, upper florets were still lost without the assistance of super glue during sectioning. Given the sequential arrangement of meiosis in florets within one spikelet, this loses valuable information for meiosis staging. In view of the drawbacks of vibratome sectioning, we used instead wax embedding sectioning for the experiments in this paper. Using this approach, spikelets of various sizes can be sectioned entirely from base to top without any loss of samples (Fig. 1G–K). Importantly, the final FISH results of the wax embedding sectioned samples are comparable with those of the vibratome sectioned samples.

It is common to stage plant meiosis by staining meiotic chromosomes with acetocarmine or similar dyes on anther squash samples. However, the anther squash samples of *B. distachyon* failed to be stained properly by any of the dyes tested including acetocarmine, carbol fuchsin and DAPI, although these dyes did produce strong and clear staining in parallel wheat anther squash samples (data not shown). As an alternative, *B. distachyon* meiosis was staged after cell wall digestion and DAPI staining of young thin sections from young spikelets. The meiotic stage of a given meiocyte was determined based on the chromatin morphology, sizes of the spikelets, the timing of sample collection and also the pattern of labelled centromeres and/or telomeres if available. DAPI stained meiocytes at different meiotic stages are shown in Fig. 1 B–F. In detail, meiosis starts day 25 (±2 days) after germination and the whole process from early prophase to tetrad formation lasts for approximately one day. One typical *B. distachyon* plant has several inflorescence-carrying tillers, 2–3 spikelets per rachis node, 5–10 florets per spikelet and 2 anthers per floret. Meiosis starts first in the tallest tiller, and one day later in the next two tillers. It also starts earlier in the bigger spikelet of the same rachis node. In this study, only the biggest spikelets from the three tallest tillers of the plants were used.

Wax section samples of base to top florets within one spikelet are shown in Fig. 1G–K. The meiotic processes were in general synchronised among pollen mother cells (PMCs) within the same floret. For the same spikelet, meiosis starts first in the base floret and a few hours later in the next floret and so on. For instance, if the base floret (Fig. 1G) is at pachytene, the second from base floret (Fig. 1H) would be at a stage around leptotene to zygotene, and the third from base floret (Fig. 1I) at pre-meiosis. For a tray of *B. distachyon* plants germinated and grown at the same time, meiosis is in general synchronized among the majority of the plants although it is common to see a few plants starting the process of meiosis one day prior to or after the majority. Since young spikelets are still fully covered by stems when meiosis starts in the base floret, it is hard to judge when to collect the early meiosis samples based on a single plant alone. As an alternative, newly emerged young spikelets of a few plants beginning meiosis were used as a sign for sample collection on the following day if early meiosis base floret samples were needed. Otherwise, emerged spikelets were collected for wax embedding sectioning, and early meiosis florets could be found in the upper parts of such spikelets.

### 2 Identification of Centromeric Satellite Sequence in *B. distachyon*

**Figure 2:**
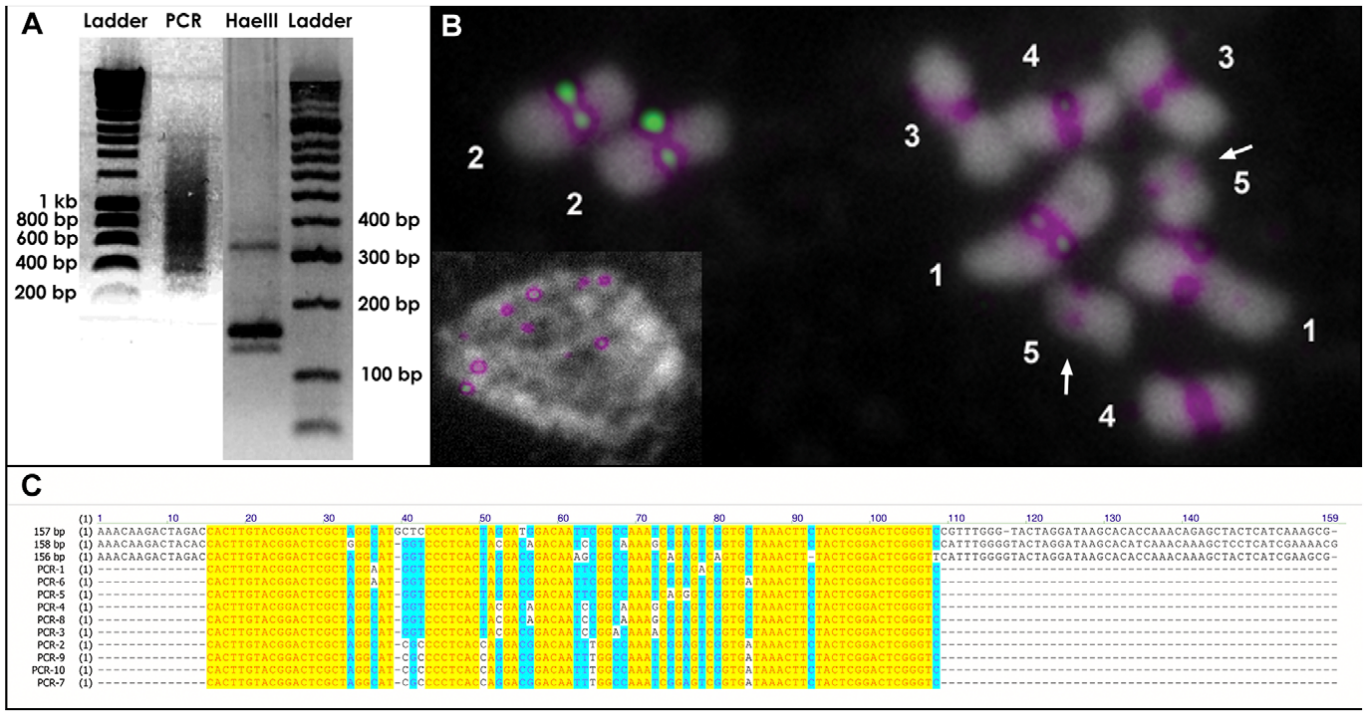
Identification of *B. distachyon* centromeric satellite sequence. **A**: PCR amplification of the presumptive *B. distachyon* centromeric satellite sequences from genome DNA, and the HaeIII digestion of the PCR product; PCR product appeared as a ladder on the agar gel with an interval of around 156 bp, which was as expected from a tandem repeat with a unit size of 156 bp. A unique HaeIII recognition site was found within the monomers of most presumptive *B. distachyon* centromeric satellite repeats. After the full digestion with HaeIII, the PCR product mentioned above appeared as three bands on the agar gel. A strong band was located at approximately 156 bp, with another slightly lower, faint. These two bands represent satellite monomers of two different sizes, with the major species the 156 bp one. There is another faint band located at about 310 bp, which probably represents mutated HaeIII recognition sites in a small portion of satellite monomers (arrow). **B**: FISH labelling of the presumptive *B. distachyon* centromeric satellite sequences on a metaphase chromosome spread. The intensity of labelling was indicated by a green-purple intensity lookup table (purple representing weaker labelling, and green representing stronger labelling). The labeled probe sequences were specifically located at the centromeres of all chromosomes, which confirmed these sequences as the true centromeric satellite repeat sequences. The labelling also shows that the abundance of the *B. distachyon* centromeric satellite (CentBd) repeats varies significantly among different chromosomes, with chromosome 5 (arrow) containing the smallest amount of CentBd. **C**: Alignment of presumptive *B. distachyon* centromeric satellite sequences with 10 actual sequences of PCR products. As shown by the picture, the presumptive sequences and actual PCR product sequences were highly similar with each other, which suggested that the PCR products were indeed amplified from the presumptive satellite sequences.

Most plants, including Brachypodium, share an identical 7 bp telomere tandem repeat sequence (TTTAGGG)_n_. The protocol for labelling such a telomere sequence is well established. Brachypodium telomeres were successfully labelled in preliminary experiments using this sequence. However, the centromere probe CCS1 that was previously used in our group to label cereal centromeres failed to label Brachypodium centromeres in the preliminary experiments (data not shown). The fully assembled genome sequence of *B. distachyon* was not available until early 2010 [15], and the centromeric sequences were not identified at the time of this study. Centromeric satellite repeats are the most repeated sequences in the centromeric region, and therefore are often used as the probe in the FISH labelling of centromeres. To identify the centromeric satellite repeat sequence of *B. distachyon*, the reported cereal centromeric sequences CCS1 (Genebank ID: U52217.1 [26]) and pSau3A9 (Genebank ID: U68165.1 [27]) were used to BLAST against the 4× coverage preliminary genome sequence assembly at www.modelcrop.org with default setting to identify the centromeric sequence segments containing both homologues of CCS1 and pSau3A9. Two such segments (super_2:2420000–2430000 10 kb, super_3:21200002–21250000 50 kb) were obtained at www.modelcrop.org. A bio-informatics tool (Phobos, Ver 3.2.6) was used to search for tandem repeat sequences within these two segments. Three long tandem repeat sequences with good scores were found in the super_3 segment. Interestingly, these three sequences were highly similar to each other both in size (156 bp, 157 bp, 158 bp respectively) and in sequence (Fig. 2C). Consensus sequence of these three was used to BLAST against the 4× coverage preliminary genome sequence assembly again. Highly conserved sequences of these three were found in many super-contigs with a uniform pattern as expected for centromeric satellite repeats. These 156 bp sequences were therefore suspected to be the centromeric satellite repeat sequences of *B. distachyon.*

To confirm that the identified candidate sequence was truly the centromeric satellite sequence of *B. distachyon*, PCR primers selected from the conserved regions were used to amplify the candidate centromeric satellite sequence from *B. distachyon* genomic DNA. The PCR products appeared as a DNA ladder on a gel with an interval of about 156 bp as expected (Fig. 2A). After fully digesting the PCR products with HaeIII restriction enzyme, one strong band and two faint bands were found. The strong band was located at approximately 156 bp, with one faint band slightly lower. These two bands represent satellite monomers with two different sizes with the most abundant being that at 156 bp. There was another faint band located at about 310 bp, which probably represented the mutated HaeIII recognition sites in a small proportion of the satellite monomers (Fig. 2A). The PCR products were then cloned and sequenced. Sequencing data showed that the PCR products were highly conserved with the suspected satellite sequence, and had the same pattern of conserved and variable regions.

To determine whether these sequences were located in centromeric regions, the PCR products were used to label metaphase chromosomes by FISH. The signals specifically covered centromeric regions of all the five chromosomes (Fig. 2B), and thus the identified tandem repeats were confirmed as the centromeric satellite sequence of *B. distachyon* and named as CentBd. It was also found by the FISH labelling that the abundance of the CentBd repeats varied significantly among different chromosomes, with chromosome 5 containing only a tiny amount of CentBd (Fig. 2B).

### 3 Centromere and Telomere Behaviour during Early Meiosis

Telomeres (in green) and centromeres (in red) were labelled by FISH on thin tissue sections (10–30 µm) to examine chromosome behaviour during the process of chromosome pairing in *B. distachyon*. *B. distachyon* is a diploid (2n = 10). Considering the tiny centromeres that chromosome 5 has (Fig. 2B), unassociated centromeres would be expected to be labelled as 8 bright dots plus 2 fainter dots, paired centromeres as 4 bright dots plus 1 fainter dot, and clustered centromeres (with more than two centromeres associated as one cluster) as less than 4 bigger dots. The faint labelling of chromosome 5 centromeres may or may not be detectable depending on the FISH sensitivity.

**Figure 3:**
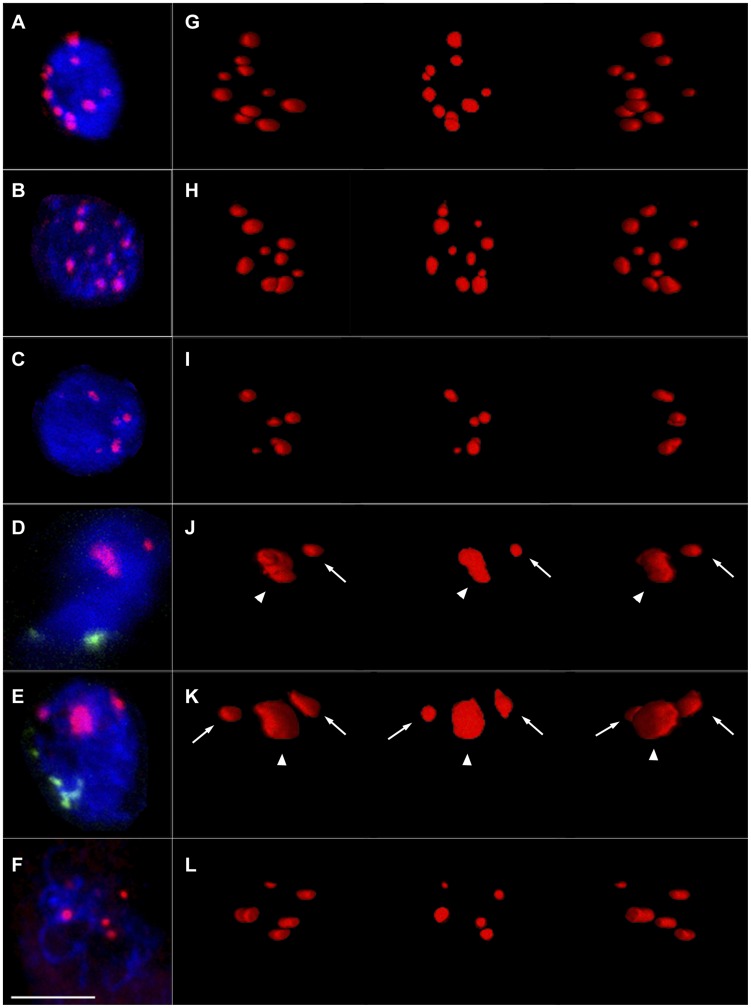
*B. distachyon* centromere behaviour during early meiosis. **A-F** : Confocal maximum projection images of *B. distachyon* PMC nuclei (DAPI; blue) at different early meiosis stages, labelled with centromeres (A-F; red) and telomeres (D, E; green). Bar = 5 µm. **G–L:** 3D maximum intensity projection images of centromeres in the corresponding nuclei from **A–F**. In each image of **G–L**, −30°, 0° and +30° y axial rotation projection images of the same reconstruction are shown from left to right in sequence. **A, G**: A typical anther somatic nucleus with 10 normal size and dispersed centromere dots. Centromeres were not associated in anther somatic nuclei at any stages. **B, H**: A typical pre-meiotic PMC nucleus with 10 normal size and dispersed centromere dots. Centromeres were not associated in most pre-meiotic PMC nuclei. **C, I**: A late pre-meiotic PMC nucleus with 5 normal size centromere dots, which shows the pairing of centromeres in some late pre-meiotic PMC nuclei that are about to go through meiosis. **D, J**: A typical leptotene PMC nucleus with one centromere block (**J**, arrow head) and one normal size centromere dot (**J**, arrow), which shows that centromeres are clustered at this stage. **E, K**: A typical zygotene PMC nucleus with one centromere block (**K**, arrow head) and two normal size centromere dots (**K**, arrow), which shows that the clustering of centromeres was maintained during this stage. **F, L**: A typical pachytene PMC nucleus with 5 normal size centromere dots. Centromere clusters were resolved as paired centromeres at this stage.

**Figure 4:**
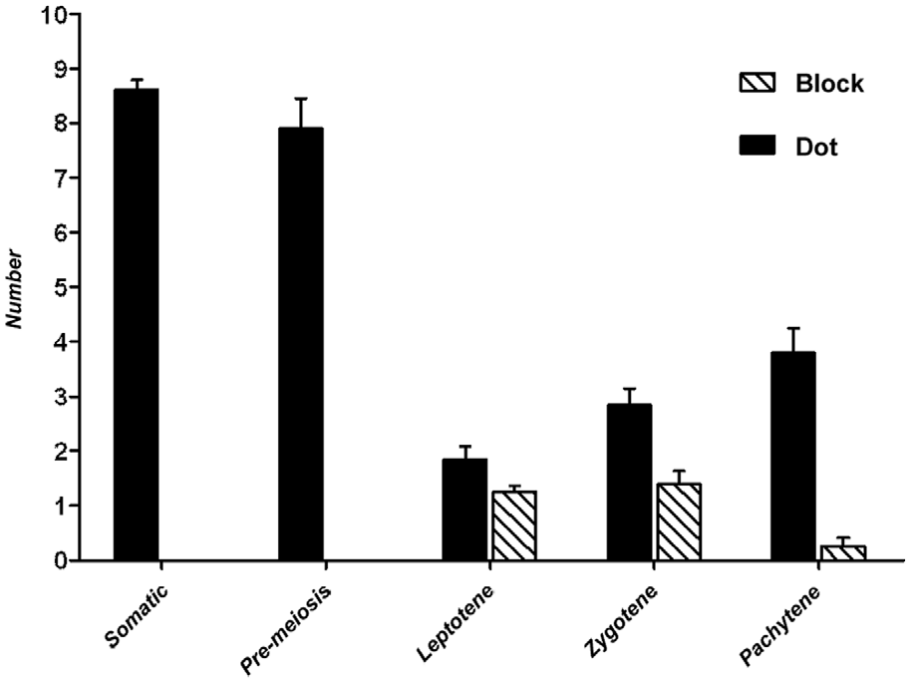
Diagram summarising the centromere number of anther cells at different early meiosis stages. Bar represent the SEM. Centromere number in the pre-meiosis PMC cells (7.9±0.6) was nearly the same as in the anther somatic cells (8.6±0.2, P = 0.124 in one-tail t test); A centromere clustering event which brought down centromere dot number to 1.8±0.2 occurred at leptotene stage with the emergence of centromere blocks (indicated by arrow heads in **Fig. 3. J, K**); Centromere block number was maintained during zygotene (1.2±0.1 to 1.3±0.2, P = 0.737 in two-tail t test), but centromere dot number was increased to 2.8±0.3; Centromere blocks almost disappeared (0.3±0.2) at pachytene, and centromere dot number was further increased to 3.8±0.4, which is just about half of the centromere number in the anther somatic cells (8.6±0.2).

The results are shown at Fig. 3 and summarised in Fig. 4. As these results show, centromeres were not associated and were randomly dispersed across the whole nucleus in most of the pre-meiotic PMCs (7.9±0.6 dots, n = 17, Fig. 3B, H) as well as in the surrounding somatic cells (8.6±0.2 dots, n = 46, Fig. 3A, G) of all stages. Pre-meiosis is a vaguely defined stage that contains PMCs at different stages with some of them closer to the onset of meiosis than others. Due to the lack of defined pre-meiosis markers, it is hard to sub-stage pre-meiosis precisely. However, it is possible to compare the ages of pre-meiotic PMCs of different florets by comparing the stages of their adjacent older florets within the same spikelets. It was noticed that, very rarely, centromeres appeared as pairs in the late pre-meiotic PMCs (Fig. 3C, I), and this kind of centromere association in PMCs was never found in PMCs at early pre-meiosis. A significance test to compare the average centromere numbers at pre-meiosis PMCs and surrounding somatic cells (7.9±0.6 compared to 8.6±0.2, P = 0.124 in one-tail t test) showed that centromere number was not significantly reduced in the pre-meiosis PMCs. The results altogether suggested that centromeres remain unassociated during the long period of pre-meiosis, and only started to be paired near the transition point of pre-meiosis to meiosis.

The PMC centromeres showed dramatic changes around the onset of meiosis, when PMCs were changing from pre-meiosis to leptotene. During this narrow time period (less than 2 hr), the average number of centromere dots reduced (possibly via a brief paired configuration as described above) from 7.9±0.6 to 1.8±0.2 (n = 37), which was coupled with the emergence of an unusual centromere organisation that appeared in the form of large blocks (Fig. 3J, K; arrowheads). The results indicated that a centromere clustering event bringing separated centromeres into blocks occurred at the start of meiosis. As meiosis progressed, the average number of centromere dots per PMC increased from 1.8±0.2 to 2.8±0.3 (n = 24) and then to 3.8±0.4 (n = 16) when PMCs went through the stages of leptotene to zygotene and then to pachytene. Meanwhile, the average number of centromere blocks was nearly maintained from leptotene to zygotene (1.2±0.1 to 1.3±0.2, P = 0.737 in two-tail t test), but almost disappeared (0.3±0.2) later at pachytene. The average centromere dot number in a pachytene PMC (3.8±0.4) was about half of the average centromere dot number in anther somatic cells (8.6±0.2), which indicated that centromeres were fully paired at pachytene. Dual labelling of telomeres and centromeres showed that the centromere clustering event and emergence and maintenance of centromere blocks occurred at the same time as the formation and maintenance of telomere bouquet structures (Fig. 3D, E).

## Discussion

### 1 The Canonical Arrangement of Cereal Core Centromeric Sequences is also Found in *B. distachyon*

Cereal centromeric sequences, like other plant centromeric sequences, are mainly composed of centromeric satellite sequences and transposable elements (TEs). In cereals, the first identified centromeric sequences were CCS1 [26] and pSau3A9 [27]. Both CCS1 and pSau3A9 are parts of a specific Ty3/gypsy retrotransposon family that are commonly referred to as the cereal centromeric retrotransposons (CCRs) [28], [29]. Members of CCR were found to be present in the centromeric regions of various cereal genomes with highly conserved motifs [30] and TEs within the cereal centromeric region are mainly members of this CCR family [30], [31], [32]. Unlike CCRs, which are conserved among cereal species, the sequences of cereal centromeric satellites have diverged significantly between species from different cereal genera. Nevertheless, a given species normally has only one type of centromeric satellite sequence and monomers of the same centromeric satellite type are in general highly conserved [31], [32], [33]. Interestingly, variation of centromeric satellite monomers tends to appear at specific sites rather than randomly [31], [32]. It was reported that in *A. thaliana* the distributions of the dominant centromeric satellites pAL1 and the major centromeric retrotransposon Athila are distinct, with pAL1 mainly located at core centromeric regions and Athila at pericentromeric regions respectively [34], [35]. The fact that pAL1 is the only major centromeric DNA binding CENH3 in a CHIP assay [36] suggested that the centromeric satellite is the only dominant core centromeric sequence in *A. thaliana*. However in cereals, both CCR and centromeric satellites are crucial components of core centromeres. It was found by deep sequencing and alignments of several cereal centromeric regions, that a typical cereal core centromere contains both centromeric satellite tracts (tandem repeats of satellite monomers) and clusters of TEs that separate the satellite tracts [30], [31], [32]. Also both centromeric satellite and CCR bind strongly to CENH3 in the CHIP assay, with no obvious difference in affinity. It has been suggested that the unique composition of cereal core centromere sequences represents their status as newly formed centromeres, which is consistent with the finding that active genes exist in the centromeric regions of rice chromosomes [37].

**Figure 5:**
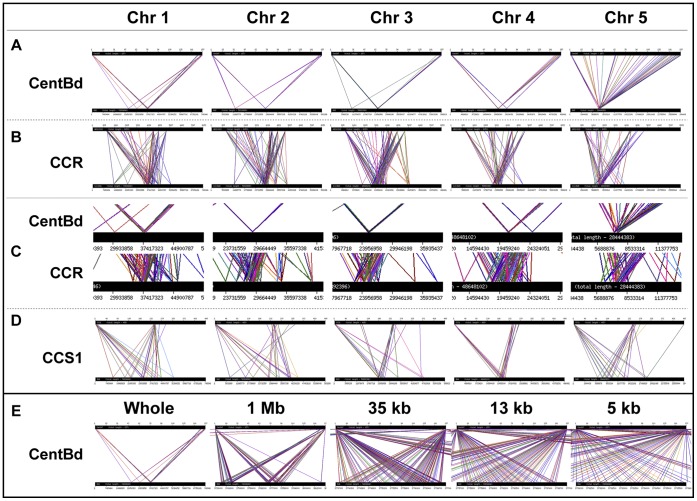
Bioinformatic analysis of *B. distachyon* centromeric regions. Centromeric sequences were BLASTed against the assembled *B. distachyon* genome sequence to show their distribution within the genome. Each small picture shows the BLAST result of one particular centromeric sequence against one particular chromosome. In each small picture, the upper bar represents the full length of the query sequence and the lower bar represents the full length of one chromosome. The left and right borders of one homologous domain are linked by a pair of lines of the same color. **A**: Distribution of CentBd homologous sequences on each chromosome. CentBd homologous regions were enriched at a few loci on each chromosome, with one major locus per chromosome containing thousands of CentBd homologous regions. **B**: Distribution of *S. bicolor* CCR homologous sequences on each chromosome. On each chromosome, CCR homologous sequences were enriched in large numbers at one major locus. **C**: Magnified images of **A** and **B** at the same narrow centromeric region of each chromosome. The CentBd enriched loci were surrounded by an enrichment of CCR homologous sequences. **D**: Distribution of CCS1 homologous sequences on each chromosome. The CCS1 homologous sequences are comparatively less abundant. CCS1 homologous regions were enriched at the centromeric region of chromosome 3 and 4, but were more dispersed on chromosome 1, 2 and 5. **E**: Serial magnified pictures of the major CentBd enriched locus of chromosome 1. The major CentBd enriched locus contains two CentBd blocks that are 300 kb from each other. The CentBd blocks are about 100 kb in size and are formed by tandemly arranged CentBd monomers.

The assembled whole genome sequence of *B distachyon* was exploited to assess the organisation of CentBd and other CCR sequences. This enabled us to determine if the CentBd sequence identified in this study is arranged in the *B. distachyon* centromeres in a similar manner to other cereal centromeric satellite sequences in their core centromeres. As shown at Fig. 5A, B, both *Sorghum bicolor* CCR homologous sequences (Fig. 5B) and CentBd sequences (Fig. 5A) are specifically enriched at the centromeric regions of each chromosome with a high abundance. It is also observed in magnified images (Fig. 5C) that, within each of *B. distachyon* centromeres, most CentBd repeats are concentrated at one narrow locus (about 100 kb) and surrounded by CCR homologous sequences that cover a much wider range (about 5 Mb) on each centromere. Serial magnified images of one CentBd enriched region are shown in Fig. 5E. These images show that CentBd repeats are further separated into smaller tracts within the region, and the entire blocks are composed of tandemly arranged CentBd repeats. The *B. distachyon* centromere structures are thus consistent with previously reported cereal centromere structures.

As suggested from the studies of other cereal centromere structures, the CentBd-enriched regions should approximately define the core centromere positions and the CCR covered regions define the peri-centromeric regions. The distribution of CenH3 and centromere related histone modifications would need to be assessed in order to confirm the organization of pericentromeric regions and core centromeres in *B. distachyon*. Finally, the analysis revealed that the CCS1 homologous sequences are much less abundant than those of CentBd and CCR homologous sequences in the genome of *B. distachyon*, and their centromere specific enrichment was not obvious on chromosomes 1, 2 and 5 (Fig. 5D). This observation raises a question as to the importance of CCS1 as a centromeric sequence in *B. distachyon*. This contrasts with the great abundance of CCS1 in a species closely related to *B. distachyon* (*B. sylvaticum*), where it was first identified, and in other cereal centromeres.

### 2 Meiotic Centromere Clustering of B. distachyon May Act in Two Possible Ways

**Figure 6:**
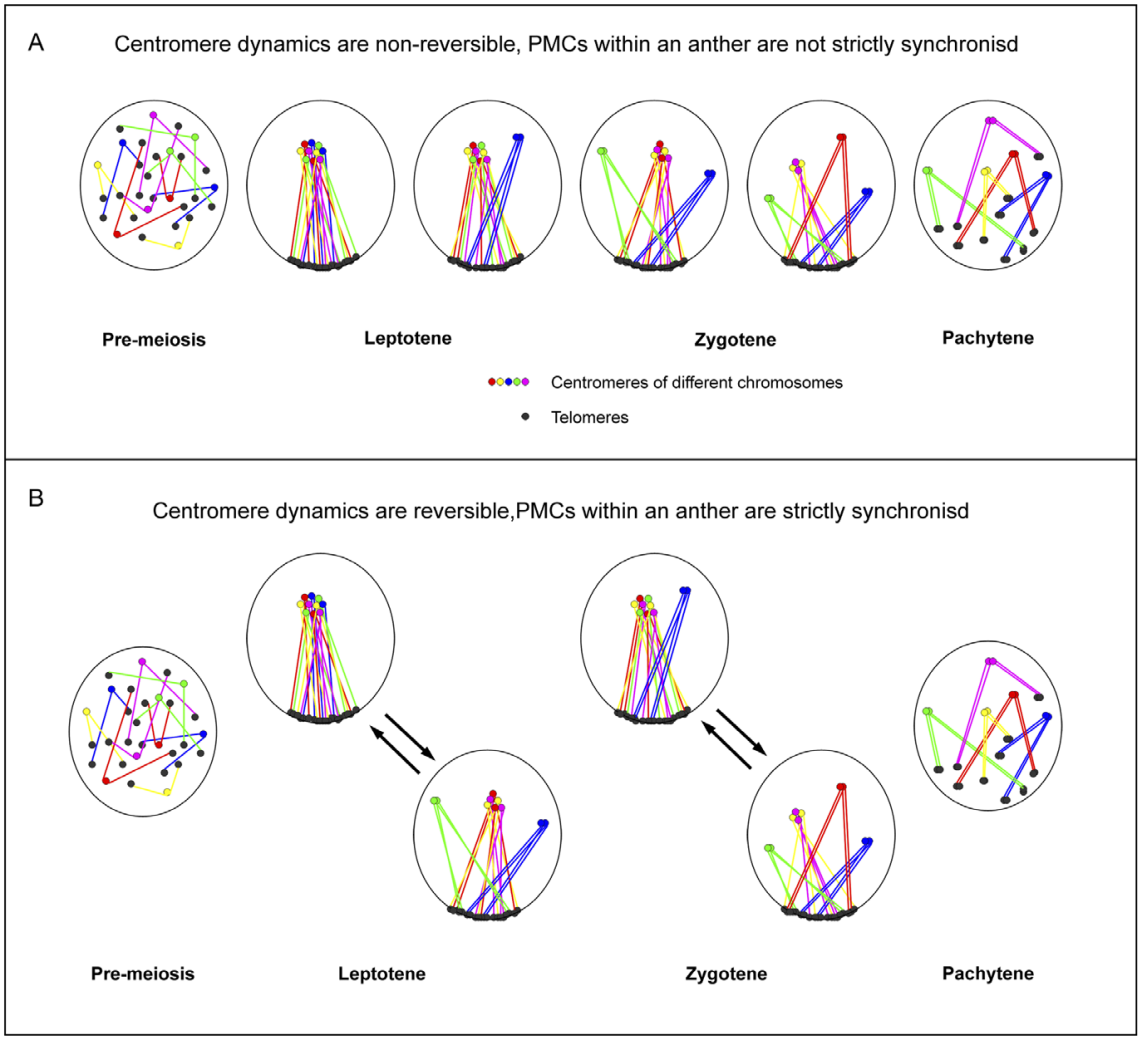
Proposed models of centromere behaviour during early meiosis of *B. distachyon*. **A**: Model of centromere behaviour during early meiosis as a one-way non-reversible process, in which centromeres cluster into one block and homologous centromeres then resolve as pairs one after another. In this case, PMCs within the same anther would not be strictly synchronised. Variable centromere patterns would represent PMCs at different time points in the process. **B**: Model of centromere behaviour during early meiosis as a dynamic reversible process, in which centromeres commute frequently between clustered blocks and paired dots; homologous centromere pairs are established gradually during the process. In this case, PMCs within the same anther would be synchronised and undergoing the same dynamic process. Variable centromere patterns would represent PMCs fixed at different phases of the same dynamic process.

The numbers of centromere clusters varied among PMCs derived from the same anther during early meiosis, with a few extreme PMCs exhibiting only one large centromere cluster. However such extreme examples were only seen in leptotene anthers but not in zygotene anthers. Presumably the behaviour of all centromeres in *B. distachyon* PMCs is controlled by the same mechanism and they follow the same process. The variation in PMC centromere patterns (different numbers of clusters) at early meiosis can be explained by two possible models (Fig. 6).

In the first model the centromeres are undergoing a one-way, irreversible process, in which centromeres form into one cluster and homologous centromeres resolve as pairs from this cluster; variable centromere patterns would then represent PMCs at different time points of this process. PMCs within the same anther would not be strictly synchronised in this case (Fig. 6A). The alternative model is that centromeres are undergoing a dynamic and reversible process, in which centromeres commute rapidly and frequently between clusters and pairs or single sites, and homologous centromere pairs are established gradually during this process. In this case variable centromere patterns would represent PMCs that are fixed at different phases of the same dynamic process; PMCs within the same anther could be considered as synchronised in a dynamic process (Fig. 6B). A potentially similar process was observed with the formation of clusters during premeiotic replication in wheat-rye hybrids, when the number of centromere sites varied between 7 and 14 [38].

### 3 Similar Meiotic Centromere Behaviour in Other Species

Centromere behaviour during early meiosis has also been studied in rice, wheat and Arabidopsis [8], [11], [13], [39], [40]. These studies have shown that centromeres of polyploid wheat lines associate in pairs in the floral tissues long before the onset of meiosis. In wheat relatives, the centromeres were found to be in pairs before meiosis in the floral tissues of all the polyploids, including newly synthesized ones, but not in any of the investigated diploid wheats [11]. In contrast, centromeres were reported to be associated before meiosis in the pollen mother cells of all the investigated rice species, which include both polyploids and diploids [40]. In Arabidopsis, the centromeres associate at the onset of meiosis, and one or two centromere clusters can be observed in PMCs at early meiosis [13]. The present study shows that in the diploid *Brachypodium* species *B. distachyon* centromeres only start to pair in the PMCs at the beginning of meiosis forming a few large clusters (Fig. 3, 4), which is consistent with what was observed in Arabidopsis.

### 4 What is the Function of the *B. distachyon* Meiotic Centromere Clustering Event?

In polyploid wheat species, centromeres initially associate in random pairs, which then reassort to homologous pairs during the course of meiosis. Interestingly there is a “bridge” step linking the random to homologous centromere association status. At this “bridge” step, which is just before the onset of meiosis, centromeres cluster into 7 groups in the PMCs of both hexaploid and tetraploid wheat before resolving into paired sites [12]. In wheat-rye hybrids, the 28 centromeres, comprising the 21 wheat centromeres (7 centromeres from each of the A, B and D related genomes) and 7 rye centromeres, cluster as 7 sites at the onset of meiosis as the telomere bouquet is formed. The 28 wheat-rye centromeres initially pair during floral development as 14 sites but then sort into 7 clusters during premeiotic replication prior to the onset of meiosis [38]. During premeiotic replication, the number of centromere sites ranges between 14 and 7, before stabilizing to 7 sites. Each of the 7 centromere clusters at the onset of meiosis contains a single rye centromere, implying that the 7 clusters are composed of homoeologous centromeres. This suggests that the centromeres first associate non-homologously during floral development, then engage in a sorting process during premeiotic replication before forming 7 clusters, which resolve as homologous pairs. Corredor *et al.* [41] proposed that sorting of centromeres into homologous pairs was driven by telomere pairing; as the homologous chromosomes intimately aligned and synapsed from the telomere regions towards the centromere regions, the associations involving the centromeres switched from non-homologous to homologous. This proposal was based on the pairing of two chromosomes, one a rye chromosome and one a wheat chromosome whose centromere was replaced with a centromere segment homologous to that in the rye chromosome. These two chromosomes are unable to pair with each other via their telomere regions in wheat. Moreover the rye centromeres of these two chromosomes do not pair with each other during meiosis. This implied that homologous centromeres could not pair during meiosis when the rest of the chromosome could not pair, and therefore that telomere pairing drove centromere pairing. However the study did not show that the two rye centromeres within these chromosomes actually treated each other as “homologous” with respect to the centromere pairing process and could actually pair with each other at all. When centromeres pair during floral development, a level of homologous pairing (15%) is observed. Yet these two rye centromeres failed to pair with each other at all at this stage, implying that when a rye centromere is inserted within a wheat chromosome, its structure may be altered so that it no longer treats the corresponding centromere in the originating rye chromosome as homologous. Another possibility is that the rye centromere may have been inserted in the incorrect orientation, in which case it would be treated as non-homologous. Moreover recently, contrary to the proposal of Corredor *et al.* [41], studies in Arabidopsis have clearly shown that centromeres do pair homologously with each other independently of pairing of the chromosome arms [13]. These experiments did not use chromosome constructs, in contrast to Corredor *et al*. [41], but instead used Rad51 mutants, which eliminated chromosome pairing of the arms and telomeres, but not the centromeres. Da Ines *et al*. [13] further showed that not only can the centromeres pair homologously, but that synaptonemal complex formation can be initiated at these paired centromere sites, independently from the telomere regions. The synaptonemal complex formation then extends out from these centromere regions. This suggests that centromeres in plants can pair independently of the rest of the chromosome, and that synapsis can be initiated within these regions.

The behavior of the *B. distachyon* centromeres at the onset of meiosis is similar to that in Arabidopsis, in that the centromeres can cluster as a single site and resolve at later stages of meiosis as paired sites. The implication is that this clustering, as with the telomere bouquet, enables the centromeres to be brought into close proximity to each other, allowing a sorting process to occur in much the same way as with telomeric regions. Why would plants have evolved a centromere pairing process independent from telomeres? Plants, in comparison to mammals for example, can have very large chromosomes and carry multiple genomes, yet the time taken to complete chromosome pairing during meiosis is not that much different from an organism having a genome 1000-fold smaller. Correct segregation of homologues in gametes requires that the homologous centromeres pair correctly in order that they attach to the spindle correctly at metaphase I, and are then segregated so that there are balanced chromosome complements in the gametes. If telomere pairing drives centromere pairing, then synapsis clearly needs to be completed as far as the centromeres to pair them and achieve correct segregation. If centromere and telomere pairing are independent, then full synapsis does not need to be completed in order to achieve correct segregation, which may be important for plants with very large chromosomes, or multiple genomes. Modeling studies now suggest that for large chromosomes, telomere driven pairing is not sufficient to drive full synapsis of very large chromosomes [42].

Finally the clusters formed at leptotene and zygotene in *B. distachyon* are large in size. This may represent the decondensation of the centromere satellite chromatin that forms the block (Fig. 3J, K). It is known that a sub-telomere heterochromatin decondensation event occurs concurrently with the formation of the telomere bouquet in wheat and rye [43], and is important for the pairing of homologous or homoeologous chromosomes. It would be interesting to determine if such a sub-telomere chromatin decondensation event also occurs in *B. distachyon* and if so, whether it is related with the putative centromere decondensation. Chromatin conformation changes are known to be related to epigenetic changes. It would be interesting to determine if any of the typical centromere satellite epigenetic markers like H3K9me2 or DNA methylation are specifically modified in the centromere block.
